# Integrative transcript to proteome analysis of barley during *Ramularia collo-cygni* leaf spot development identified several proteins that are related to fungal recognition and infection responses

**DOI:** 10.3389/fpls.2024.1367271

**Published:** 2024-03-28

**Authors:** René Lemcke, Manoj Kamble, Sebastian Schneider, Michael F. Lyngkjær, Simona Radutoiu, Stefanie Wienkoop

**Affiliations:** ^1^ Department of Plant and Environmental Sciences, Copenhagen University, Frederiksberg, Denmark; ^2^ Department of Molecular Biology and Genetics, Aarhus University, Aarhus, Denmark; ^3^ Department of Functional and Evolutionary Ecology, University of Vienna, Vienna, Austria

**Keywords:** *Hordeum vulgare*, fungus, transcript to protein, pathogen infection, pathogenesis related (PR) proteins

## Abstract

**Introduction:**

*Ramularia* leaf spot (RLS) disease is a growing threat to barley cultivation, but with no substantial resistance identified to date. Similarly, the understanding of the lifestyle of *Ramularia collo-cygni* (*Rcc*) and the prediction of RLS outbreak severity remain challenging, with *Rcc* displaying a rather untypical long endophytic phase and a sudden change to a necrotrophic lifestyle. The aim of this study was to provide further insights into the defense dynamics during the different stages of colonization and infection in barley in order to identify potential targets for resistance breeding.

**Methods:**

Utilizing the strength of proteomics in understanding plant–pathogen interactions, we performed an integrative analysis of a published transcriptome dataset with a parallel generated proteome dataset. Therefore, we included two spring barley cultivars with contrasting susceptibilities to *Rcc* and two fungal isolates causing different levels of RLS symptoms.

**Results:**

Interestingly, early responses in the pathogen recognition phase of the host were driven by strong responses differing between isolates. An important enzyme in this process is a xylanase inhibitor, which protected the plant from cell wall degradation by the fungal xylanase. At later time points, the differences were driven by cultivar-specific responses, affecting mostly features contributing to the pathogenesis- and senescence-related pathways or photosynthesis.

**Discussion:**

This supports the hypothesis of a hemibiotrophic lifestyle of *Rcc*, with slight differences in trophism of the two analyzed isolates. The integration of these data modalities highlights a strength of protein-level analysis in understanding plant–pathogen interactions and reveals new features involved in fungal recognition and susceptibility in barley cultivars.

## Introduction

*Ramularia collo-cygni* (*Rcc*) is an apoplastic Dothideomycetes filamentous fungus from the family Mycosphaerellaceae that colonizes a large range of grasses and cereals (Havis and Oxley, 2008). *Rcc* grows primarily as an endophyte, but in barley has developed into a pathogenic fungus. The extent of the disease developed in barley, *Ramularia* leaf spot (RLS), varies with the cultivar (CV), isolate, and environmental conditions (Hess et al., 2011). Up to 70% yield loss has been reported in South America during a heavy RLS epidemic year on susceptible CVs (Clemente et al., 2014). *Rcc* begins its life cycle as an endophyte and then switches into a necrotrophic behavior at flowering, producing rectangular-shaped necrotic spots surrounded by a chlorotic halo on the leaves (Havis and Oxley, 2008). In the necrotrophic stage, the mesophyll collapse and aerial hyphae supporting conidiophores emerge from the necrotic lesions. *Rcc* produces a photoactive anthraquinone derivative, i.e., rubellin D, a non-host-specific toxic molecule involved in the peroxidation of fatty acids (Heiser et al., 2004). Similar to cercosporin, rubellin is a photo-activated toxin that reacts with oxygen, leading to the production of singlet oxygen or superoxide. Superoxide further produces hydrogen peroxide and hydroxyl radicals, which are responsible for lipid peroxidation and pigment co-oxidation, ultimately leading to chlorosis and necrosis (Heiser et al., 2004).

RLS disease has been managed in the field with strobilurin-based treatments, but *Rcc* is a dynamic pathogen able to rapidly adapt to resistance to fungicides (Fountaine and Fraaije, 2009). Understanding the molecular mechanism behind *Rcc*–barley interactions is necessary to provide more options for breeders and farmers alike. Recent studies utilizing comparative RNA sequencing (RNA-seq) approaches have contributed to the elucidation of the fundamental molecular mechanisms underlying the host defense processes against RLS. These studies have shown the involvement of ethylene and jasmonic acid signaling, along with the activation of the phenylpropanoid and flavonoid pathways in response to RLS, indicating typical defense processes against necrotrophic pathogens (Sjokvist et al., 2019). The early production of *p*-coumaroyl-hydroxydehydroagmatin (*p*-CHDA) and serotonin, which are both integral components in the process of cell wall fortification, has been identified at the metabolite level (Lemcke et al., 2021). Furthermore, distinct expression levels of kinases, calmodulins, and defense proteins have been described as key differences between the defense responses in sensitive and resistant CVs (Lemcke et al., 2021). Although, to date, no complete resistance has been observed in the field for RLS, the symptoms vary significantly from one CV to another (Pinnschmidt et al., 2006; McGrann et al., 2015), allowing the classification of these CVs into sensitive, intermediate, and tolerant categories. Breeding for RLS tolerance is currently done by phenotyping, and no genetic markers are available. Moreover, there is no known method for predicting the intensity of the *Rcc* bloom from one year to another, making breeding assays and disease control difficult (Dussart et al., 2020).

Recent sequencing of the *Rcc* genome in 2016 identified 11,617 gene models (McGrann et al., 2016; Mulhare et al., 2021; Matzen et al., 2024). This revealed that the *Rcc* genome encodes a low number of genes predicted to code for plant cell wall-degrading enzymes, a feature common to many endophytic, biotrophic, and hemibiotrophic fungi (Lo Presti et al., 2015). Multiple toxin-related genes with orthologs to the previously described cercosporin and HC toxin [*Cochliobolus* (*Helminthosporium*) *carbonum* toxin] were also found in the genome. A total of 150 small-secreted proteins (SSPs) were predicted based on the genome of *Rcc*, with half of them predicted to be specific for *Rcc* (McGrann et al., 2016). Out of the 150 SSPs encoded in the *Rcc* genome, seven matched *in silico* to previously described effectors produced by other fungi that induce a pathogenic status in plant hosts. Two of these where shown to induce host defenses (MgSM1 and PemG1), three, interestingly, are related to the development of appressoria and haustoria (BEC1005, BEC1040, and ACE1), one to a suppressor of plant defense responses (BEC1019), and the last one to a virulence enhancer (HopL1) (McGrann et al., 2016). The molecular events controlling the transition of *Rcc* isolates from endophytic to pathogenic association in barley are currently unknown. Identifying the molecular components behind *Rcc* aggressiveness toward barley will also open more options toward controlling RLS using targeted approaches.

In this paper, we analyzed the global proteome of *Rcc*-infected barley leaves in order to identify the proteins in two CVs infected by two *Rcc* isolates at three different time points. The CVs had different sensitivities to *Rcc*, while the two isolates showed different degrees of aggressiveness. We took advantage of having these four combinations already analyzed in depth at the transcriptome level (Lemcke et al., 2021) and performed a direct comparison of the observed molecular events at the transcript and protein levels.

## Materials and methods

### Plant growth and experimental setup

Two barley (*Hordeum vulgare*) cultivars—Fairytale (Sejet Planteforædling, Horsens, Denmark) and NFC Tipple (Syngenta Seed, Cambridge, UK; referred to as Tipple hereinafter)—were chosen for their contrasting susceptibilities as monitored in previous field studies. In addition, two fungal isolates with different disease profiles were included. The *Rcc* isolate DK05 was isolated in Denmark in 2005 from the highly susceptible spring barley cultivar Braemer and was rendered being more aggressive in RLS development. In contrast, the NZ11 isolate was isolated in 2011 in New Zealand from the susceptible spring barley cultivar Doyen.

The plant and fungal growth conditions, as well as fungal inoculations, were as previously described in Sjokvist et al. (2019). Briefly, plants were grown at 19°C in 16 h of light (300 μmol m^−2^ s^−1^) and 8 h of darkness. Plant materials were sampled for RNA-seq and proteome analysis from the same plants. Fully developed second leaves were divided into three equal-sized pieces and separately snap-frozen in liquid nitrogen.

Fungal isolates were grown on potato dextrose agar (PDA) plates supplemented with 10 μL/mL of streptomycin at room temperature (RT) in darkness. At 3 weeks prior to inoculation in plants, the fungal cultures where transferred to liquid cultures by placing three agar plugs into liquid PDA media supplemented with 10 μL/mL streptomycin. The cultures were grown likewise at RT in darkness until preparation for inoculation. Plants that were 14 days old were inoculated.

The fungal hyphae from liquid cultures were filtered, washed, and sonicated to retrieve smaller pieces. The concentration of hyphae was adjusted to 5 × 10^5^ hyphae/mL and spray inoculated with an atomizer (NS18.8/26; DESAGA, Sarstedt-Gruppe, Nümbrecht, Germany) until runoff. Directly after inoculation, the plants were kept under plastic covers for 48 h to provide relative high humidity (80%–100%) for preferential fungal growth conditions. Control plants were treated the same way, but mock-inoculated with water and Tween-20 (4 μL/mL). The plants were monitored daily for symptom development and then were sampled at 3, 7, and 12 days post-infection (dpi). The leaf segments were sampled from three different plants into one pool, and biological replicates were based on independently grown *Rcc* cultures before inoculum preparation.

### Protein extraction

Proteins were extracted as previously described (Schneider et al., 2019). Briefly, snap-frozen plant material was mixed with 10% trichloroacetic acid (TCA) in acetone and incubated for 10 min in a cold ultrasonication water bath. By centrifugation at 4,000 × *g* for 5 min, the plant material was washed with 1.5 mL ice-cold 10% TCA in acetone and 1.5 mL ice-cold 80% acetone. The pellet was air dried and the proteins extracted in a 0.8-mL extraction buffer [50 mM Tris-HCl (pH 7.5), 5 mM EDTA, 0.7 M sucrose, 1% polyvinylpolypyrrolidone (PVPP, *w*/*v*), 1 mM phenylmethylsulfonyl fluoride (PMSF), 5 mM dithiothreitol (DTT), and ddH_2_O] and 0.8 mL phenol. After centrifugation at 10,000 × *g* for 5 min, the protein-containing phenol phase was transferred into a new tube. Proteins were precipitated overnight with 8 mL ice-cold 0.5% β-mercaptoethanol in acetone. The precipitated proteins were centrifuged for 10 min (4,000 × *g* at 4°C). The protein pellet was washed twice with 100 mM ice-cold ammonium acetate in methanol and twice with 80% ice-cold acetone with intermediate centrifugation (20,000 × *g* at 4°C for 5 min), dried in a vacuum concentrator, and stored at −80°C until further use.

### Digestion and LC-MS/MS analyses

The protein pellets were digested and analyzed as previously described in Turetschek et al. (2017). Briefly, the pellets were dissolved in urea buffer (8 M urea, 50 mM HEPES, pH 7.8) and quantified with the Bradford assay. For each sample, 20 µg protein was digested with Lys-C [1:100 (*v*/*v*), 5 h, 30°C] (Roche, Mannheim, Germany) and trypsin beads [1:10 (*v*/*v*), overnight, 37°C] (Applied Biosystems, Darmstadt, Germany). The sample was acidified with formic acid (FA) and loaded on stage tips (Pierce™ C18 Tips). The peptides were washed four times with FA, eluted with 0.1% FA in methanol, and stored at −80°C in a protein LoBind tube until measurement.

The peptides were dissolved in 2% acetonitrile (ACN) and 0.1% FA and loaded in random order into a C18 column (15 cm × 50 mm column, 2 mm particle size; PepMap R RSLC, Thermo Scientific, Waltham, MA, USA) via an Ultra HPLC (Thermo Fisher Scientific, Bremen, Germany) for separation during a 90-min gradient at a flow rate of 300 µL min^−1^. Measurement was performed on an LTQ-Orbitrap Elite (Thermo Fisher Scientific, Bremen, Germany) with the following settings: full scan range, 350–1,800 *m*/*z*; maximum, 20 MS2 scans [activation type, collision-induced dissociation (CID)]; repeat count, 1; repeat duration, 30 s; exclusion list size, 500; exclusion duration, 60 s; charge state screening, enabled with rejection of unassigned and one charge states; minimum signal threshold, 500.

### Protein identification

The barley proteome of UniProt *H. vulgare* 189799, downloaded November 2019 (ID: UP000011116), and 160517_Hv_IBSC_PGSB_r1_proteins_HighConf_REPR_annotation fasta (downloaded from e!DAL—Plant Genomics & Phenomics Research Data Repository, IPK Gatersleben, Germany) were utilized. The UniProtKB *Ramularia* sequences were also tested initially, but did not retrieve any matches.

Identification and quantification were conducted using MaxQuant 1.6.5.0 with the following parameters, as previously described (Turetschek et al., 2017): first search peptide tolerance of 20 ppm; main search tolerance of 4.5 ppm; FTMS MS/MS match tolerance of 0.6 Da; maximum of three variable modifications in the oxidation of methionine and acetylation of the N-term; maximum of two missed cleavages allowed; and best retention alignment function was determined in a 20-min window and identifications were matched between runs in a 0.7-min window.

A false discovery rate (FDR) cutoff of 0.01 [at peptide spectrum match and the protein level] was set with the aid of a reverse decoy database. A minimum of six amino acids were required for the identification of a peptide, and at least two different peptides were required for the identification of proteins. The label-free quantification (LFQ) intensities were used for quantification. The mass spectrometry proteomics data have been deposited to the ProteomeXchange Consortium via the PRIDE (Perez-Riverol et al., 2022) repository with the dataset identifier PXD039541.

### Mapping of proteins to transcripts

The transcriptome dataset has been previously published by us (Sjokvist et al., 2019; Lemcke et al., 2021) and was used here for integrative analysis. To link the proteome and transcriptome data, each protein was mapped to the corresponding barley transcript identifier (Ensembl Plants) according to putative paralogue grouping by matching the identifiers from the input source (Stare et al., 2017).

### Statistics

The proteins were functionally classified with Mercator v4 (Lohse et al., 2014) and GO-molecular using UniProtKB. The data processing and statistics for the proteome and transcriptome analyses were computed in RStudio (2022.07.2) and COVAIN (Sun and Weckwerth, 2012). Protein identifiers were matched against the transcript data. Only those protein groups that matched a transcript were selected and combined with the respective transcript (**Supplementary Table S1**). Proteins that have been unambiguously identified by proteotypic peptides are marked in **Supplementary Table S2**. The proteins/transcripts present in all replicates of at least one group were statistically assessed. If less than half of the observations in a group were missing, the values were estimated via the *k*-nearest neighbor algorithm; otherwise, a minimum value (half of the lowest value multiplied by a random value between 0.1 and 1) of the respective protein/metabolite was imputed.

A two-way ANOVA was used to examine the main effects and the interactions of biotic stress (with/without pathogens) and CVs (Fairytale and Tipple) along the time course (3, 7, and 12 dpi). Differences between treatments were compared using Tukey’s multiple range test, and statistical significance was defined at *p* ≤ 0.05 and fold change (FC) ≥2 (**Supplementary Table S2**).

## Results

### Matching of the protein IDs to transcripts

Out of the 1,551 identified protein groups, 1,299 (84%) were matched against 39,734 RNA-seq transcripts. This combined dataset of 2,598 protein and RNA targets (**Supplementary Table S1**) was used for multivariate statistics. From these, 893 targets were found to be significantly changed [ANOVA: Tukey’s test, Benjamini–Hochberg (BH) corrected *p* ≤ 0.05, FC ≥ 2) either between time points, treatment, and/or CVs (**Supplementary Table S2**).

In the following, these abbreviations will be used: for cultivars, F for Fairytale (susceptible) and T for Tipple (tolerant); for the *Rcc* isolates, D for DK05 (aggressive) and N for NZ11 (mild).

### Pattern recognition using principal component analysis and hierarchical clustering

**Figure 1:**
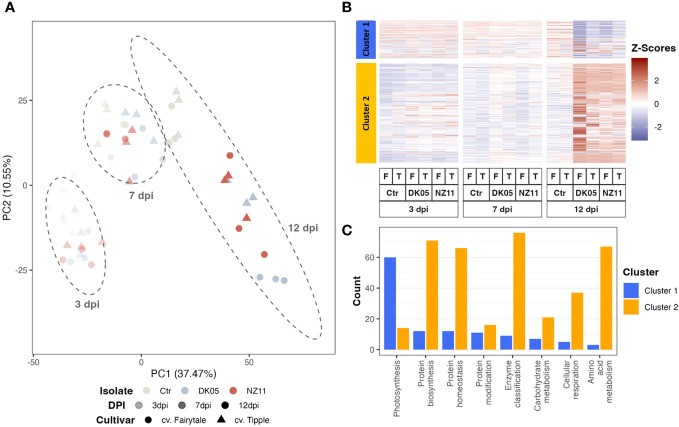
Pattern recognition across all proteins and respective transcripts: **(A)** Integrated principal component analysis. **(B)** Hierarchical cluster analysis. **(C)** Cluster distribution of the major functional groups (if >25 protein or transcript counts, excluding those not assigned). *log10*, transformed data; *dpi*, days post-inoculation; *ctr*, control. *Ramularia collo-cygni* (*Rcc*) isolates: *NZ11* mild and *DK05* (aggressive). Cultivars: *T*, Tipple (tolerant); *F*, Fairytale (susceptible).

Principal component analysis (PCA) and hierarchical clustering analysis (**Figure 1A**) were used to visualize the strongest separation of time point 12 dpi (including controls) from all earlier time points on PC1 (37.4%), while PC2 explained some minor (10.5%) separation between time points, treatments, and controls.

The hierarchical cluster analysis (**Figure 1B**) indicated two major clusters, of which cluster 1 separated the proteins and transcripts that decreased upon pathogen treatment and/or along 12 dpi, while the larger cluster 2 mostly linked those compounds that accumulated with time and/or infection (**Figure 1B**). The major enriched functional group in cluster 1 was involved in photosynthesis (**Figure 1C**). Mainly the transcripts of this major group decreased significantly (ANOVA: *p* ≤ 0.05, FC ≥ 2) at 12 dpi (**Figure 1**). However, the protein levels showed mainly only decreasing trends. In fact, at 7 dpi, the protein levels even appeared to accumulate, in contrast to their transcripts. **Supplementary Figure S1** shows the box plots of the average relative intensity levels of the 12 major RNAs (with statistically significant increases in all CVs and treatments at 12 dpi compared to the controls) belonging to photosynthesis (**Figure 1C**, cluster 1) and their respective proteins, which were not changed significantly.

Cluster 2 included protein regulation (synthesis and homeostasis) as well as amino acid metabolism and cell respiration among the main groups (**Figure 1C**). These major groups in clusters 1 and 2 naturally represent the major classes of the whole dataset (see enrichment plot, **Supplementary Figure S2**). However, the class “protein modification” was enriched compared to the overall enrichment, while photosynthesis was depleted in cluster 2.

### Extraction of major stress-responsive targets (external stimuli) and correlation analysis

**Figure 2:**
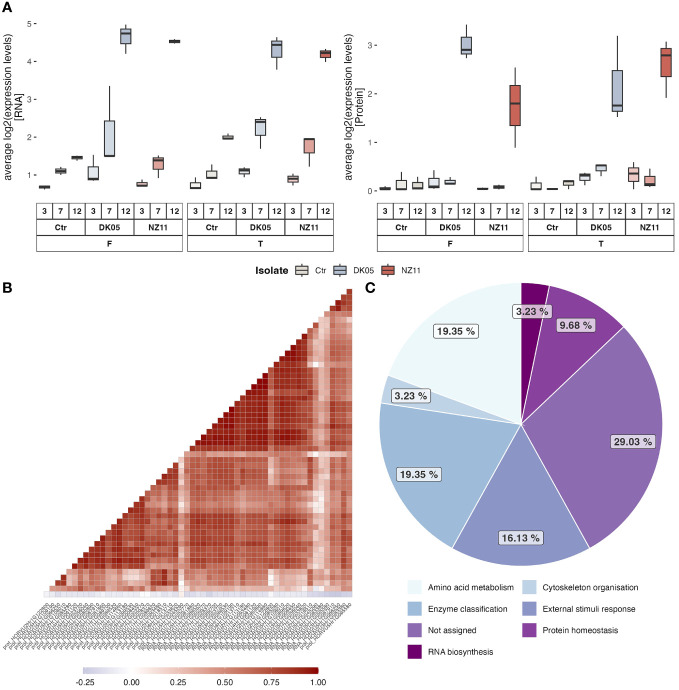
In-depth analysis of the major late stress-responsive features (12 dpi). **(A)** Box plots of the average levels of RNA and proteins with the highest loadings in PC1 (**Supplementary Table S3**). **(B, C)** Correlation analyses of the respective RNA and proteins **(B)** and their distribution of functional categories **(C)**.

After the extraction of the highest loadings from **Figure 1** (PC1 loading >0.05) (**Supplementary Table S3**), the average levels of the 30 targets (transcripts and respective proteins) were plotted (**Figure 2**; separate box plots can be found in **Supplementary Figures S3A–D**). The single plots for each ID can be found in **Supplementary Figure S3**. These transcripts and/or proteins showed a significant (*p* ≤ 0.05, FC ≥ 2) increase in abundance at 12 dpi in at least one of the CVs/treatments after pathogen infection (**Supplementary Table S2**). Most of these high loadings were transcripts, in accordance with their commonly higher relative abundance values compared to the protein levels. However, correlation analyses of the respective target proteins showed overall good correlation (**Figure 2B**). The membrane protein (HORVU7Hr1G052770) did not correlate positively to its respective transcript (the protein was also not in the highest loadings and showed rather early response signals in Tipple). The main functional categories of these PC1 targets that separated treatments and controls differed in functional enrichment compared to the major overall functional categories. **Figure 2C** shows that the external stimulus response (stress response targets) was now one of the main groups (19%) and therefore significantly enriched compared to the overall enrichment (<2%) (**Supplementary Figure S2**) and compared to the other functional groups. Furthermore, the RNA and protein levels of treatment FD (Fairytale, DK05, at 12 dpi) were the highest on average, while the protein levels of FN (Fairytale, NZ11, at 12 dpi) appeared the lowest (**Figure 2A**). It is worth noting that three of the high-loading transcripts (i.e., HORVU3Hr1G083380, HORVU5Hr1G095580, and HORVU3Hr1G067910) (**Supplementary Figure S3D**) showed increased levels at 12 dpi also for the controls.

### Extraction of specific cultivar, pathogen, and time point relation patterns

**Figure 3:**
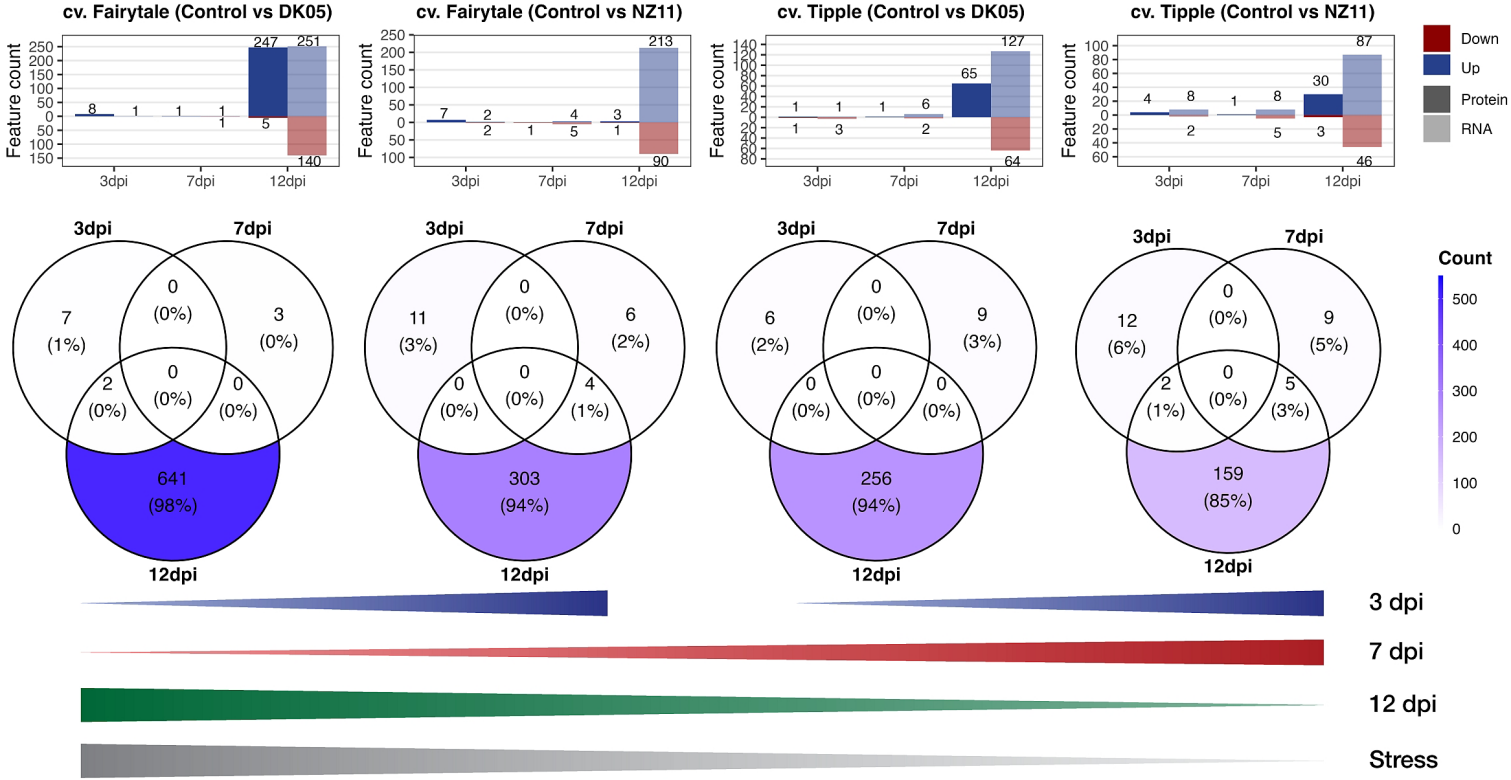
Venn diagrams. *Upper panel*: Number of proteins (*P*) and transcripts (*T*) significantly (*p* ≤ 0.05, FC ≥ 2) changed (up or down) from the respective controls (see also **Supplementary Table S2**). The Venn diagrams show overlaps of the significantly changed P and T. *Lines* (*lower panel*): Schematic summary of the increasing or decreasing numbers of P and T at respective time points related to the level of “stress” according to Lemcke et al. (2021). *dpi*, days past-inoculation. *NZ11* (mild) and *DK05* (aggressive) denote the *Ramularia collo-cygni* (*Rcc*) isolates.

Comparison of the significantly changed transcripts and proteins (against the control levels) revealed overlapping and differential patterns between time points and pathogen treatments of the two CVs (**Figure 3**). At 3 and 7 dpi, in general, a low number of features (transcripts and proteins) were found (maximum, 14), while the largest group of changes was detected at 12 dpi independent of CV and pathogen. In addition, the majority of features showed increased levels during stress, in accordance with cluster 2 (**Figure 1B**). The numbers of responsive transcripts and proteins were highest in FD > FN > TD > TN, corresponding to the previously reported infection levels (Lemcke et al., 2021). While most of these features were transcripts, about 50% of the features in FD were proteins. The earlier time points showed a different pattern; in fact, 7 dpi showed the exact opposite trend, with the highest numbers from TN > TD > FN > FD (**Figure 3**). Small overlaps at 7 and 12 dpi were detected for pathogen *Rcc* isolate NZ11 (mild). At 3 dpi, significant features were found higher with pathogen NZ11, corresponding to the PCA patterns, where 3 dpi showed separation among pathogen treatments, but not CVs. In contrast, the later time points showed more pronounced CV clusters (**Figure 1A**). In addition, 7 dpi showed the lowest numbers of features compared to 3 and 12 dpi in both CVs. Altogether, Fairytale (susceptible) showed the highest number of significantly changed features (i.e., 655 and 325) after pathogen treatment compared to Tipple (tolerant) (i.e., 271 and 187). In addition, Fairytale showed some early responses; for instance, HORVU3Hr1G006440, a xylanase inhibitor, was annotated to external stimuli response and was not significant for Tipple compared to the control. Furthermore, both plants treated with pathogen DK05 (aggressive) showed higher numbers of significantly changed features (i.e., 655 and 271) compared to the controls and pathogen NZ11 (i.e., 325 and 187). Furthermore, there were targets that showed increasing transcript levels already in the controls at 12 dpi (**Supplementary Figure S3D**), which were to be distinguished from pathogen-related targets. These targets did not show significant differences compared to the controls, but only to other time points.

## Discussion

### Early-stage fungal recognition

We recently profiled the global transcriptomes of the two CVs, Fairytale (susceptible) and Tipple (tolerant), and found the largest differences between those at the early stages of fungal colonization (Lemcke et al., 2021).

While transcriptomics allows for the early detection of the fungal pathogen in barley leaves before the appearance of disease symptoms (Mäe et al., 2018), it is interesting to analyze how this translates into proteins and whether variations in the protein synthesis and turnover dynamics affect pathogenicity and influence susceptibility. Altogether, changes in the transcript levels are more sensitive, but may not represent actual cell activity and protein levels. Here, we focused on those transcripts for which we now also identified proteins and found that most of the early-stage responsive targets of the previous study, mostly cell wall and membrane proteins, were not among them. This may partly be due to our extraction of soluble proteins, hence the hydrophobic proteins being not abundant in our dataset or below detection for mass spectrometry. However, membrane proteins, such as receptors and kinases, are key proteins for early pathogen pattern recognition and defense and might therefore be more abundant in the tolerant CVs (Tang et al., 2017). Therefore, membrane protein enrichment would be interesting in future studies, similar to the one we published on *Pisum sativum* membrane protein responses to the pathogen *Didymella pinodes* (Desalegn et al., 2016).

In this study, we found an interesting stress response-related candidate that showed a significant increase early at 3 dpi only in the susceptible Fairytale. HORVU3Hr1G006440 is a xylanase inhibitor whose expression is often induced in response to a pathogen attack (Tundo et al., 2022). When a plant detects the presence of pathogenic organisms, it can upregulate the production of xylanase inhibitors as part of its defense response. This helps the plant resist the enzymatic degradation of its cell walls by inhibiting the pathogen’s xylanases. Here, this indicates that Fairytale recognized *Rcc* early compared to the tolerant Tipple. Especially in the early stages after infection, when the plant needs to recognize the pathogen, it is remarkable that the difference in the response between pathogens was stronger than that between CVs. The ability of a plant to distinguish between biotrophs and necrotrophs is very likely conserved and not a CV-specific trait (Trdá et al., 2015). However, at later time points, this changes, and our data revealed more differences between CVs irrespective of the intruder.

### High correlation of the PR transcript and protein regulation at the later stages of infection

Pathogenesis-related (PR) genes/proteins have been reported to be key players in pathogen defense (Linthorst and Van Loon, 1991). They are therefore important targets in order to understand pathogen resistance. In accordance with our previous study (Sjokvist et al., 2019), at the later stage (12 dpi), several targets were found (**Table 1**), which were assigned to methyltransferases and PR transcripts/proteins and were well correlated. PR proteins accumulated not only locally in the infected but also in uninfected tissues of plants and are usually associated with salicylic acid signaling at the early stage of infection (Jain and Khurana, 2018). Interestingly, statistically, the proteins were lagging behind the transcripts, which suggests a possible delay or inhibition in the translation of these transcripts. However, this delay was less pronounced in the case of Fairytale and DK05. Fairytale has previously been demonstrated to be the more susceptible CV (Lemcke et al., 2021). Moreover, pathogen DK05 is the more aggressive strain (Lemcke et al., 2021). The higher translation rates along more significant protein accumulations in the most severely stressed CV indicate that most of the targets we selected are involved in necrotic interactions, or possibly indicating susceptibility rather than resistance. This is supported by other studies, such as that by Lambertucci et al. (2019), which showed that a PR5 thaumatin-like protein was required for susceptibility toward powdery mildew in the epidermis and the extrahaustorial membrane of barley leaves. It also revealed the need to uncover the mechanism that bypasses the controlled protein biosynthesis and the possible repression of the PR proteins and their possible posttranscriptional regulation, in future. This hypothesis is supported by the fact that the proteins involved in modification increase with time.

**Table 1:** Putative susceptibility marker for Rcc.

Identifier	Mercator category	Description
HORVU1Hr1G093480	Amino acid metabolism	Put. tryptophan synthase alpha chain
HORVU2Hr1G080890	Amino acid metabolism	Glutamine amidotransferase type 1 domain containing protein
HORVU2Hr1G113180	Amino acid metabolism	D-3-phosphoglycerate dehydrogenase (EC 1.1.1.95)
HORVU4Hr1G002270	Amino acid metabolism	5-Methyltetrahydropteroyltriglutamate--homocysteine S methyltransferase (EC 2.1.1.14)
HORVU4Hr1G061120	Amino acid metabolism	Anthranilate synthase (EC 4.1.3.27)
HORVU7Hr1G114660	Amino acid metabolism	IGPS domain-containing protein
HORVU1Hr1G089520	Enzyme classification	Put. methyltransferase
HORVU1Hr1G089620	Enzyme classification	Put. methyltransferase
HORVU1Hr1G089700	Enzyme classification	Predicted, put. methyltransferase
HORVU3Hr1G099530	Enzyme classification	Put. Cytokinin-N-glucosyltransferase
HORVU2Hr1G085280	External stimuli response	Chitin-binding type-1 domain-containing protein
HORVU5Hr1G023720	External stimuli response	Bet_v_1 domain-containing protein
HORVU5Hr1G056040	External stimuli response	Put. pathogenesis-related protein
HORVU5Hr1G106010	External stimuli response	Put. pathogenesis-related protein
HORVU5Hr1G109190	External stimuli response	Germin like (GLP), secreted, extracellular space, apoplast
HORVU6Hr1G013710	External stimuli response	Similar to secretory PR protein
HORVU2Hr1G018510	not assigned	Put. peroxidase
HORVU2Hr1G122800	not assigned	Put. heat shock 70-kDa protein
HORVU7Hr1G028370	not assigned	put. Pheophorbide a oxygenase
HORVU7Hr1G033620	not assigned	put. Cysteine-rich venom protein
HORVU3Hr1G006450	Protein homeostasis	Peptidase A1 domain-containing protein
HORVU5Hr1G081860	Protein homeostasis	put. Chaperone protein
HORVU5Hr1G045290	RNA biosynthesis	put. ethylene-responsive transcription factor

Some of the high stress-responsive targets we identified were already putatively assigned to PR proteins, which led to an enrichment of the category “external stimuli.” Nevertheless, most of these still lack functional annotation (uncharacterized/unassigned). Since the transcripts support the protein targets (or rather the other way around), these accessions can be assigned as PR-related (**Table 1**).

Other than the previously reported RNA levels in Fairytale (Lemcke et al., 2021), which were low at the later stages, in this study, the majority of the features showed increased levels during stress. This was also true for the more resistant Tipple (**Figure 1**, cluster 1). Interestingly, the few targets that showed increased protein (and transcript) levels only in Tipple were similarly high initially in Fairytale, but did not show a change in their levels, e.g., HORVU5Hr1G106010 and HORVU2Hr1G018510. In this case, the higher protein levels could indicate regulation mechanisms for actual enhanced *Rcc* resistance, such as inhibition of protein degradation. These are good targets for further investigation.

Moreover, the targets with increasing transcript levels already in the controls at 12 dpi indicate a general senescence process that occurs over time, not related to pathogen-induced stress. Previously, we found evidence of a typical phytohormone signaling response to necrotrophic pathogens, which involved jasmonate and ethylene, when exposing barley to the aggressive *Rcc*, DK05 (Sjokvist et al., 2019). These results indicate that the *Rcc* isolate DK05 is probably not biotrophic but switches to necrotrophic behavior, indicative of a hemibiotroph. Higher or faster responses at the beginning of infection, similar to priming, could also have an advantage in reducing pathogenicity. In the current study, a clear difference was observed in the early stage of infection (3 dpi) between the two pathogens independent of cultivar. NZ11 (a mild *Rcc*) appeared to cause a stronger response (more significant changes) in the early phase of infection in both CVs compared to DK05 and showed milder responses later on. These results confirm the previous hypothesis that the strains differ in their trophic interaction with the plant. Further studies are needed to, for example, detect salicylate levels at the early stages of infection.

The large group of photosynthesis-related transcripts with decreasing levels compared to proteins indicates that photosynthesis is not closely regulated at the transcript level and/or that protein degradation (but also synthesis) is strongly delayed compared to the decrease in RNA levels. Data suggest that the protein levels may decrease at the later time points, i.e., when proteins might degrade possibly through posttranslational regulation, reduced synthesis, and enhanced senescence effects. It has been shown that proteins do not necessarily break down when the RNA levels decrease, e.g., after stress release, but remain accumulated (Schneider et al., 2019). It is also possible that the induction of protein synthesis is delayed and the levels do not increase simultaneously with the transcript levels, as shown for the Rubisco large subunit of *Chlamydomonas reinhardtii* along a diurnal cycle (Recuenco-Muñoz et al., 2015). In summary, these data demonstrate that there is no clear correlation between the transcript and protein levels upon stress for the functional category of photosynthesis regulation.

Taken together, while PR protein responses can be analyzed well at the transcript level, RNA-sequencing does not appear to be a useful tool to analyze the effects of *Rcc* infection on the levels of the proteins involved in photosynthesis. Fairytale showed a hypersensitive response when infected with the necrotrophic isolate DK05, underlined by a major induction of PR protein translation compared to the mild *Rcc* NZ11 and the tolerant cultivar Tipple. This was confirmed by the higher susceptibility of Fairytale (Lemcke et al., 2021). Therefore, understanding posttranscriptional regulation to reduce the translation of several enhanced PR transcripts involved in induced hypersensitive response may be a key mechanism to protect plants, especially against hemibiotrophic pathogens.

## Conclusion

In conclusion, this study provides new insights into the late-stage recognition of fungal pathogens in barley cultivars, highlighting the impact of protein dynamics on pathogenicity and susceptibility. Changes in the transcript levels appear more sensitive for early-stage fungal colonization detection, but may not reflect actual protein activity. In the early stages of infection, the responses of barley differed more between the *Rcc* isolates than the CVs. Later on, the CV differences became more pronounced. We observed a high correlation between the PR transcript and protein regulation in the later infection stages, suggesting their involvement in hypersensitive response and susceptibility. Similarly, several targets of amino acid metabolism, especially methyltransferases, were found. Moreover, we identified targets involved in protein modification that increase with time, indicating a need to investigate posttranscriptional regulation mechanisms in future studies. Our data confirm an overall higher pathogen response in the susceptible Fairytale compared with the tolerant Tipple, with some proteins showing early recognition. Notably, the *Rcc* isolates exhibited different infection behaviors, with the less aggressive NZ11 leading to early responses and the more aggressive likely necrotrophic DK05 to more intense responses at the later stages, strengthening the hypothesis that this isolate is hemibiotrophic. In conclusion, our protein-level analysis enhances our understanding of plant–pathogen interactions in barley, providing valuable insights into molecular mechanisms and a list of potential protein targets related to fungal recognition and susceptibility.

## Data availability statement

The datasets presented in this study can be found in online repositories. The names of the repository/repositories and accession number(s) can be found below: https://www.ebi.ac.uk/pride/archive/, PXD039541.

## Author contributions

SW: Conceptualization, Data curation, Formal analysis, Funding acquisition, Investigation, Methodology, Project administration, Writing – original draft. RL: Formal analysis, Investigation, Methodology, Visualization, Writing – original draft. MK: Investigation, Methodology, Writing – review & editing. SS: Methodology, Writing – review & editing. ML: Conceptualization, Writing – review & editing. SR: Conceptualization, Funding acquisition, Writing – original draft.
