# Supplementary material for: Integrative transcript to proteome analysis of barley during Ramularia collo-cygni leaf spot development identified several proteins that are related to fungal recognition and infection responses
Source: Front Plant Sci. 2024 Mar 28;15:1367271. doi: 10.3389/fpls.2024.1367271 (PMC11007159; doi:10.3389/fpls.2024.1367271)
Supplement: Supplementary file 2 [file DataSheet_2.pdf]

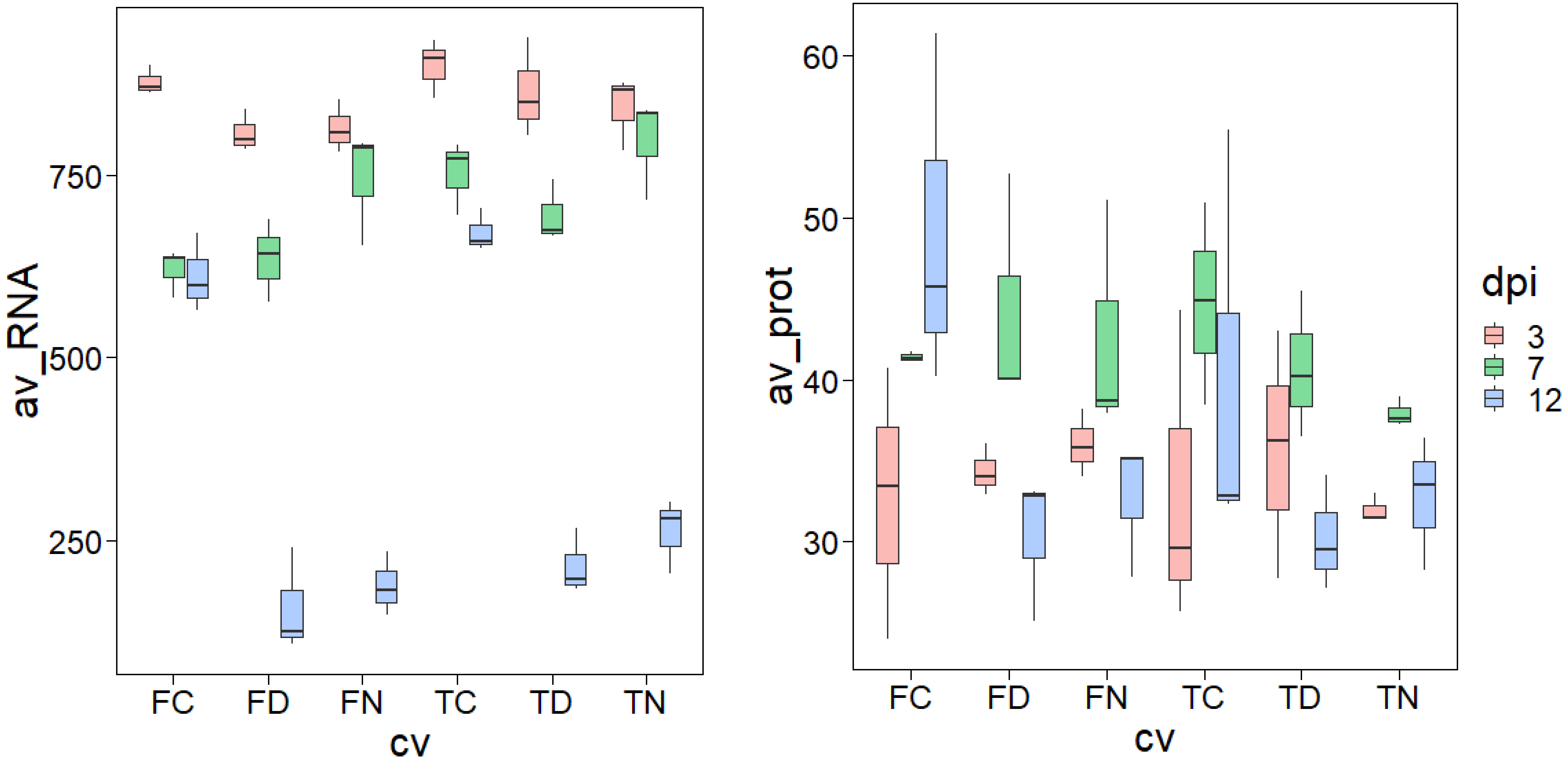

Supplemental Figure 1 Box-plots, average levels of RNA (left) and proteins (right) (overlapping significance across both CVs and pathogen treatments) of the photosynthesis (12); dpi = days past inoculation, N = path. NZ11, D = path. DK05 , T = cv Tripple, F = cv Fairytail

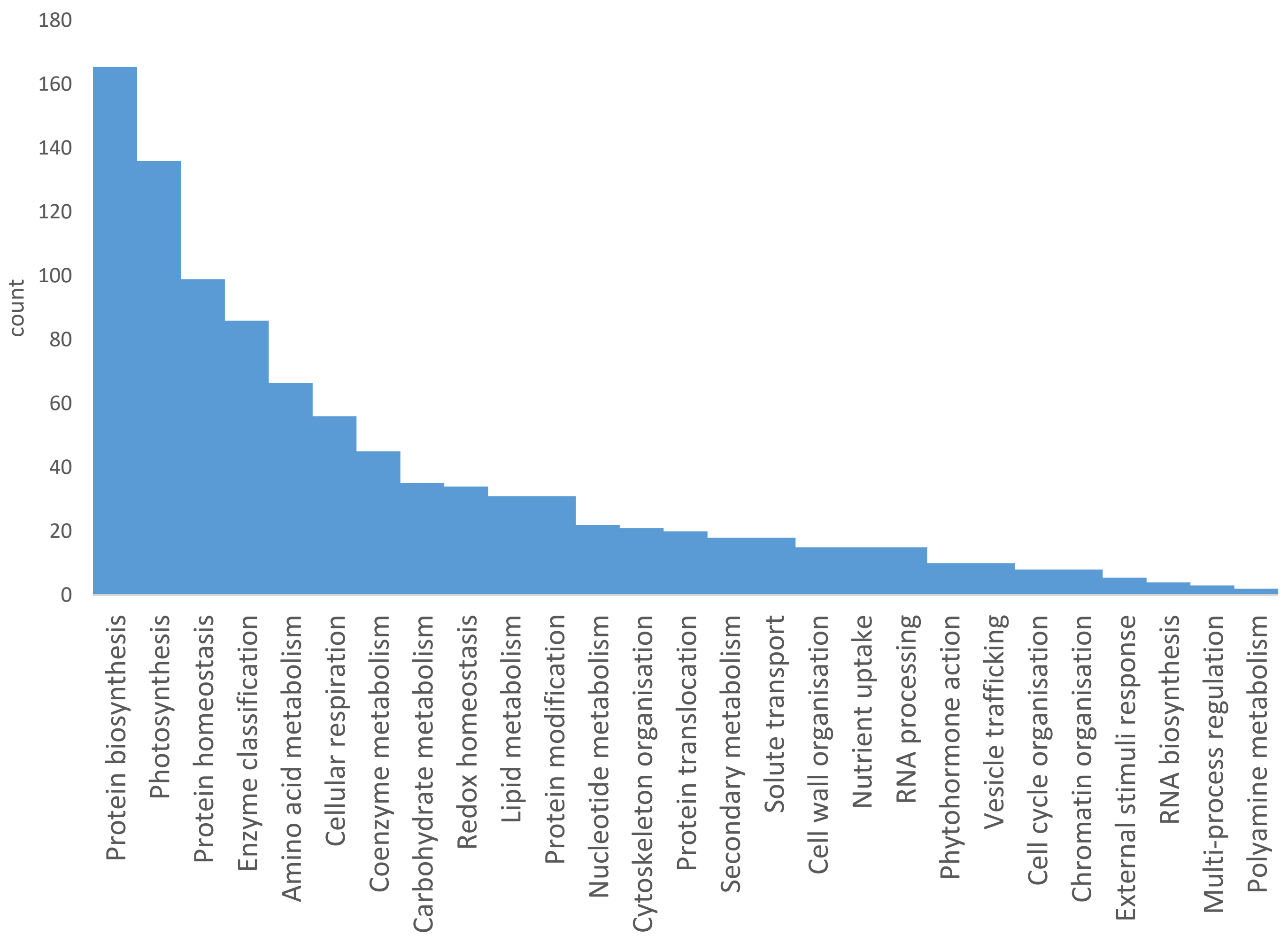

Suppl. Figure 2. Enrichment diagram (mercator) of all identified and functionally assigned proteins/RNA of Suppl. Table 1.

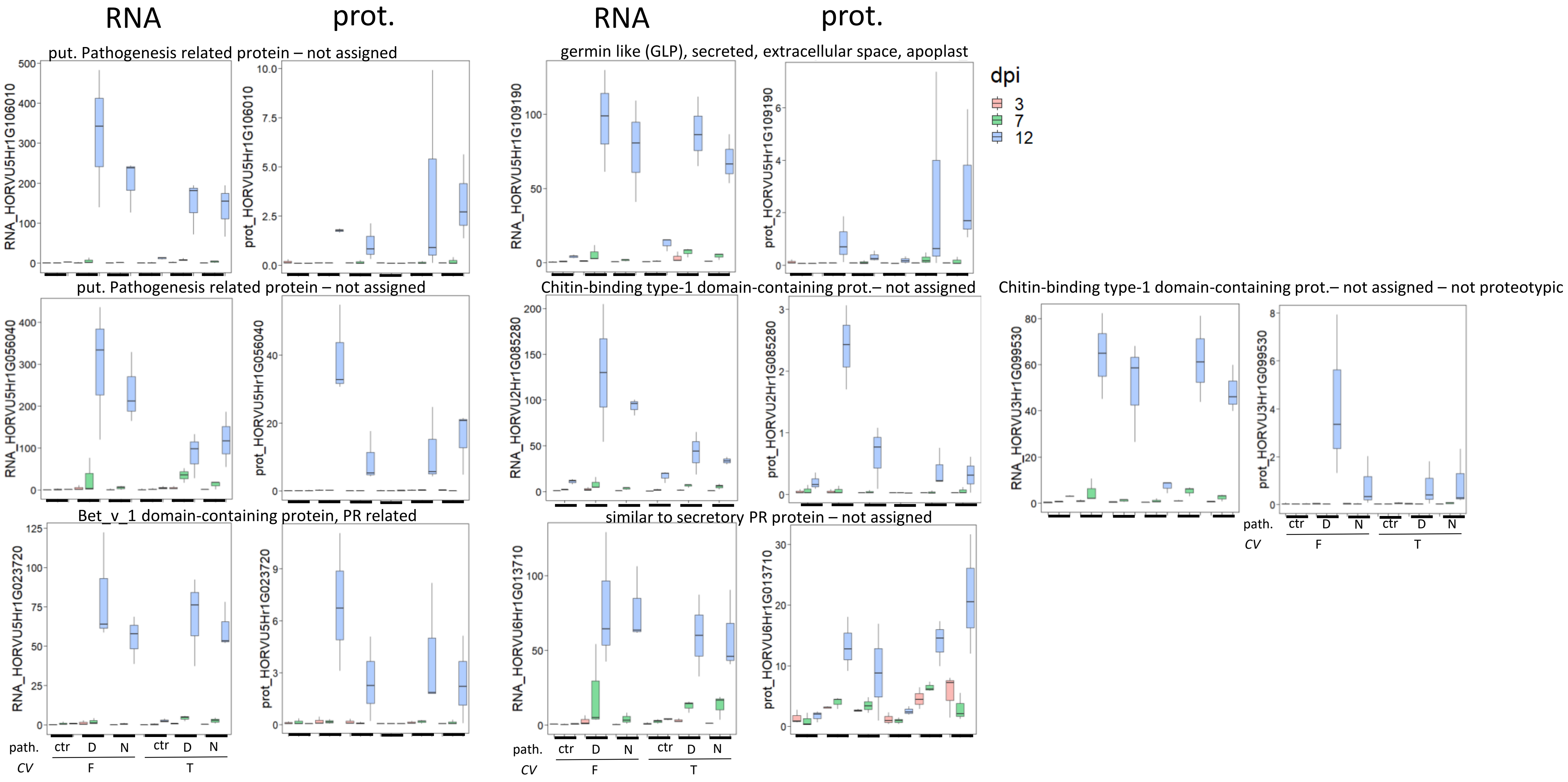

Suppl. Figure 3A Box-plots, PC1 high loadings with annotations to known PR proteins.

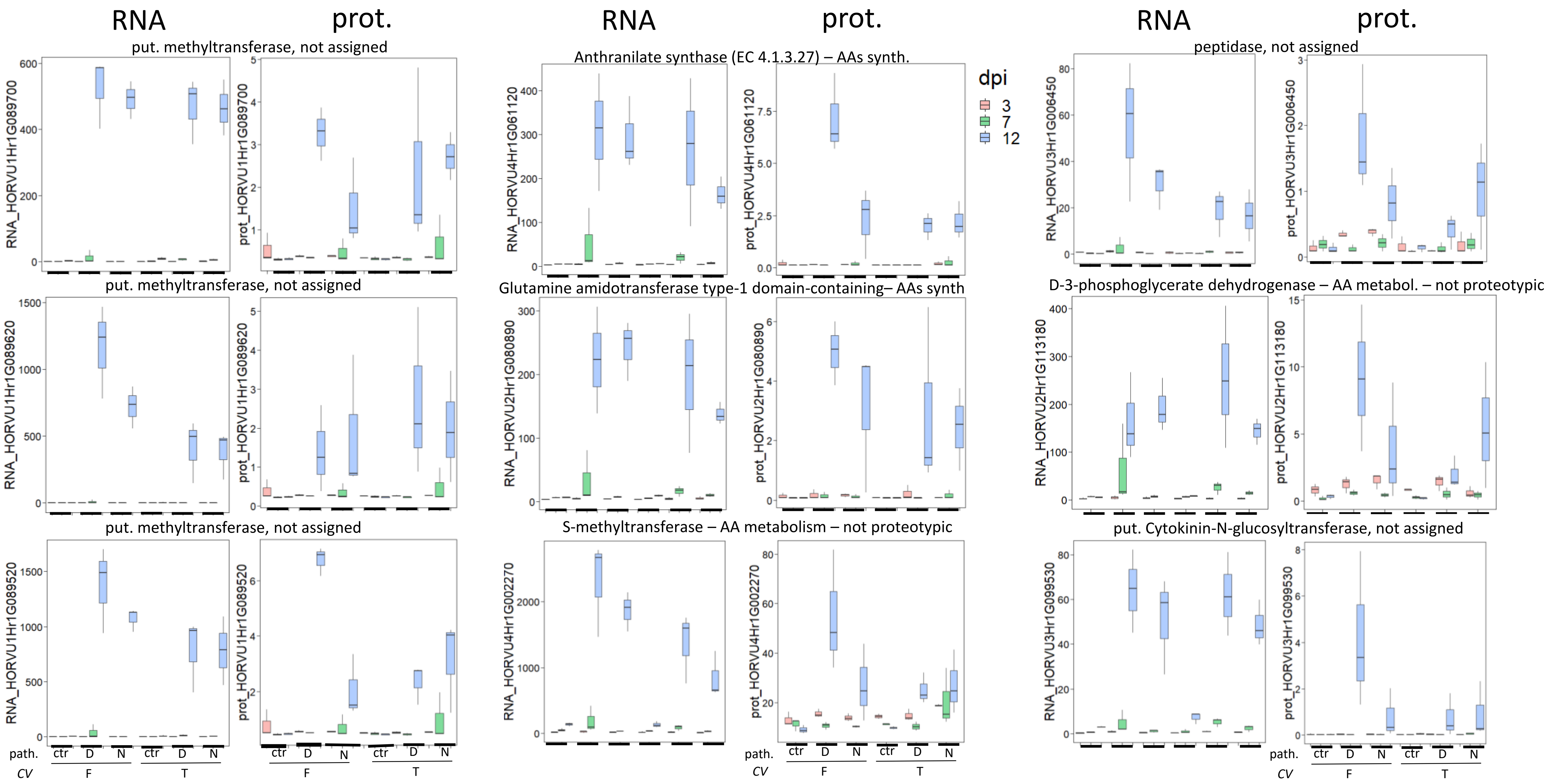

Suppl. Figure 3B Box-plots,PC1 high loadings characterized as methyltransferases or assigned to AA metabolisms.

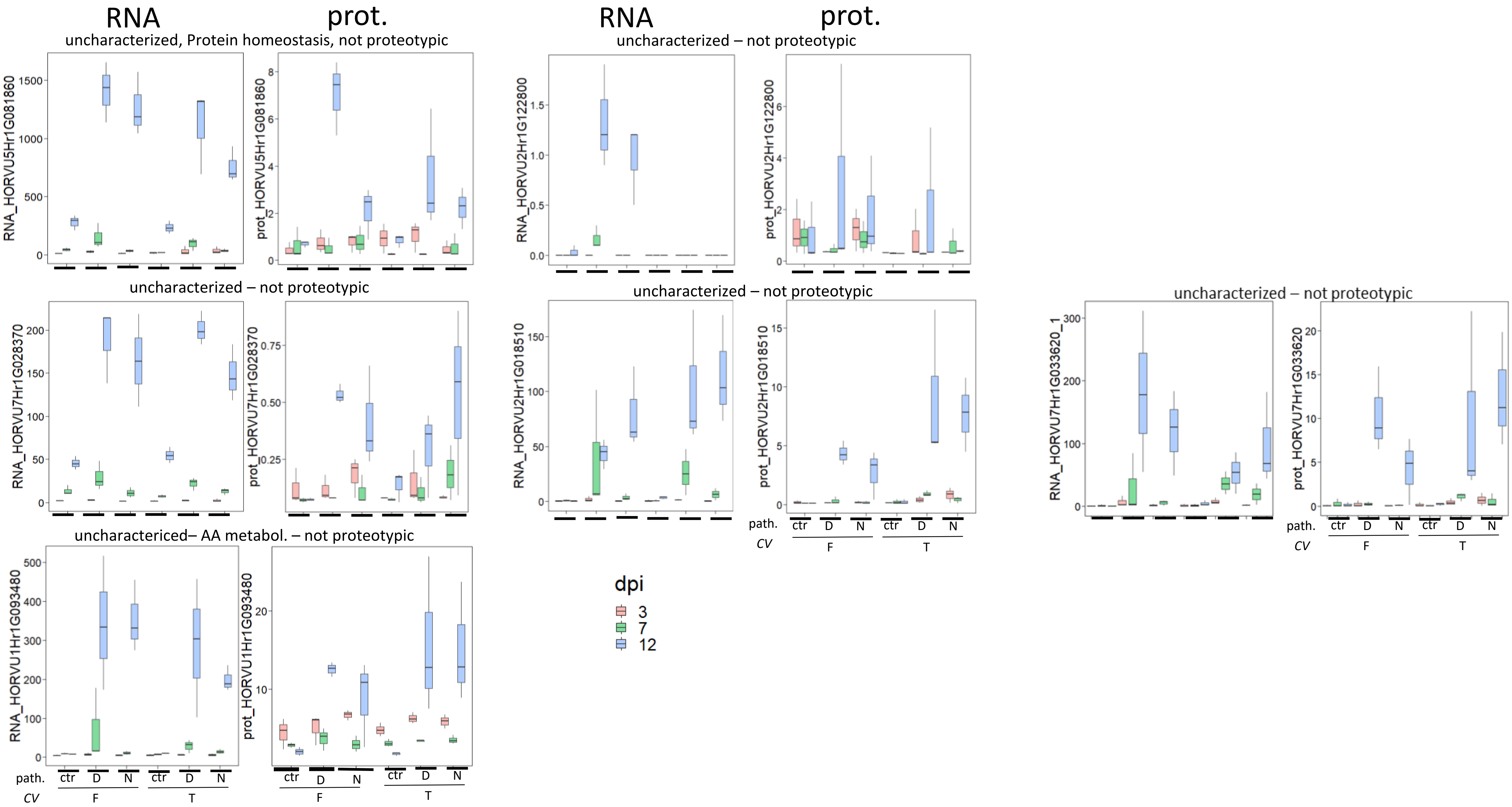

Suppl. Figure 3C Box-plots ,PC1 high loadings uncharacterized. proteins represented as groups –not proteotypic.

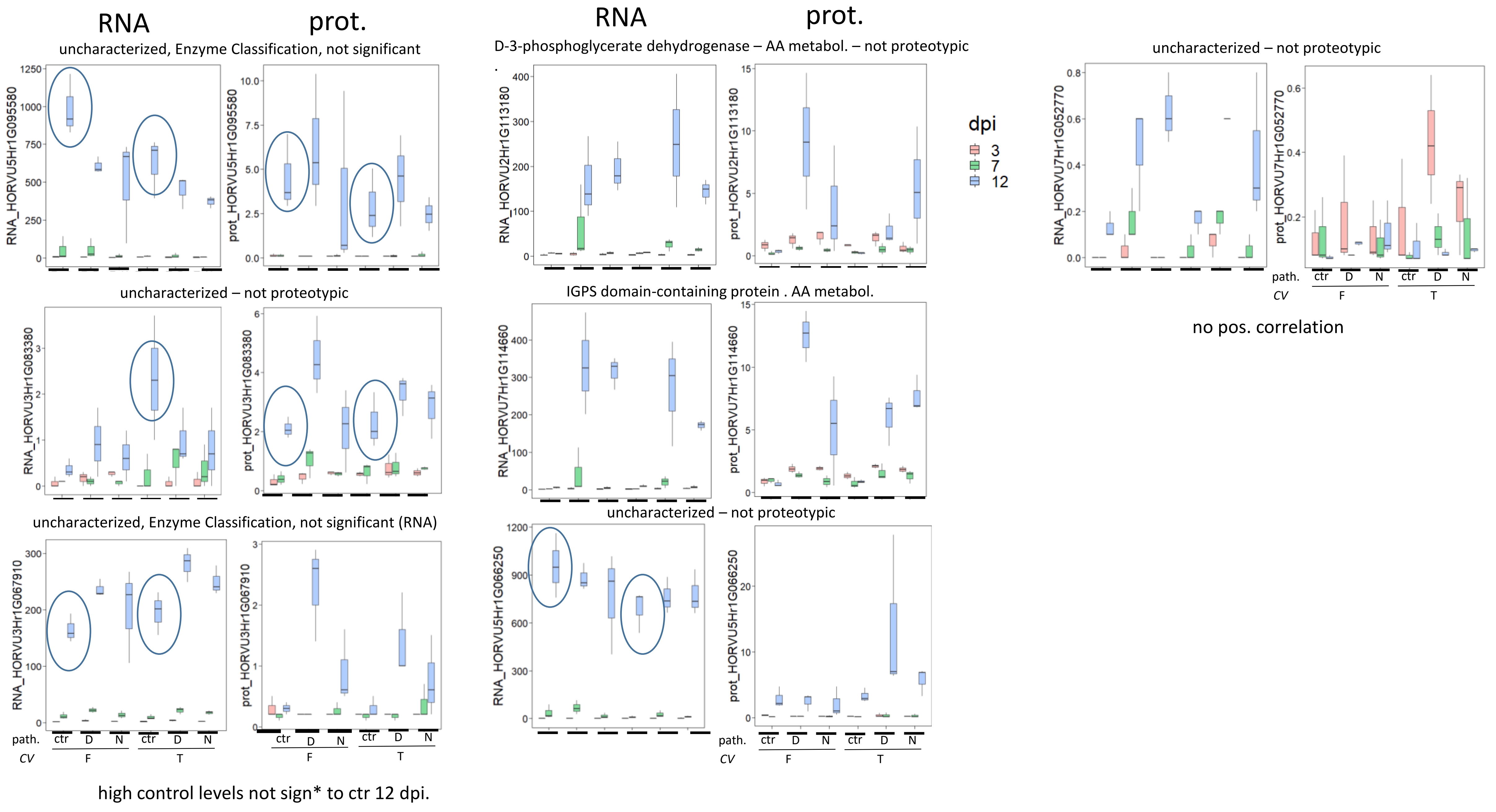

Suppl. Figure 3D Box-plots, PC1 high loadings partly assigned or uncharacterized. Circled bars indicate increased control levels.
